# The Identification of Non-Driving Activities with Associated Implication on the Take-Over Process

**DOI:** 10.3390/s22010042

**Published:** 2021-12-22

**Authors:** Lichao Yang, Mahdi Babayi Semiromi, Yang Xing, Chen Lv, James Brighton, Yifan Zhao

**Affiliations:** 1 School of Aerospace, Transport and Manufacturing, Cranfield University, Bedford MK43 0AL, UK; lichao.yang@cranfield.ac.uk (L.Y.); m.babayi-semiromi@cranfield.ac.uk (M.B.S.); Yang.X@cranfield.ac.uk (Y.X.); j.l.brighton@cranfield.ac.uk (J.B.); 2 School of Electrical and Electronic Engineering, Nanyang Technological University, Singapore 639798, Singapore; lyuchen@ntu.edu.sg

**Keywords:** non-driving related activity (NDRA) classification, level 3 automation, 3D CNN, take-over transition, situation awareness

## Abstract

In conditionally automated driving, the engagement of non-driving activities (NDAs) can be regarded as the main factor that affects the driver’s take-over performance, the investigation of which is of great importance to the design of an intelligent human–machine interface for a safe and smooth control transition. This paper introduces a 3D convolutional neural network-based system to recognize six types of driver behaviour (four types of NDAs and two types of driving activities) through two video feeds based on head and hand movement. Based on the interaction of driver and object, the selected NDAs are divided into active mode and passive mode. The proposed recognition system achieves 85.87% accuracy for the classification of six activities. The impact of NDAs on the perspective of the driver’s situation awareness and take-over quality in terms of both activity type and interaction mode is further investigated. The results show that at a similar level of achieved maximum lateral error, the engagement of NDAs demands more time for drivers to accomplish the control transition, especially for the active mode NDAs engagement, which is more mentally demanding and reduces drivers’ sensitiveness to the driving situation change. Moreover, the haptic feedback torque from the steering wheel could help to reduce the time of the transition process, which can be regarded as a productive assistance system for the take-over process.

## 1. Introduction

Conditional automation systems (level 3), defined by the SAE (J3016) Automation Levels [1], releases the driver’s eyes and hands from monitoring the environment and controlling the vehicle. Such systems can perform some non-driving activities (NDAs) during automated driving, however, they would have to intervene in the control of the vehicle when requested. Even though lots of level 2 and 3 automation systems have been commercialised in the automotive industry, the immature design and the excessive trust of the driver still cause accidents, even costing lives. Two Tesla fatalities occurred in Williston, Florida, USA, 2016 and Mountain View, California, USA, 2018. In both fatalities, the Autopilot systems were engaged, and the drivers were performing some NDAs before and when the accident happened (watching movies and playing games). Neither the Autopilot nor the driver noticed the hazard ahead and took action to avoid the accident, even though there was sufficient time and distance to react to prevent the crash [2,3]. Both fatalities could have been avoided if there was a driver monitoring and alert system to prevent the prolonged disengagement of the dynamic driving task. Since the driver’s situation awareness could be reduced and their mental demand could be increased by the NDAs engagement [4,5,6], automatically recognising the driver’s NDAs engagement and further understanding its impact on the take-over performance is of great importance to design an intelligent human–machine interface (HMI) for a safe and smooth take-over process.

In terms of NDA recognition, the most similar research is secondary task detection, which is an important part of driver behaviour monitoring for conventional vehicle safety. It aims to evaluate the degree of distraction while the driver is driving. The related methods can be roughly categorized into three groups, including vehicle maneuver-based methods, driver gesture modelling methods, and information fusion methods. The vehicle maneuver-based methods measure the vehicle state features, e.g., speed, longitudinal acceleration, lateral acceleration, pedal position, etc. Such methods provide high accuracy in terms of the engagement detection. However, the performance in terms of identifying a specific task is relatively poor [7,8]. The gesture modelling methods directly model the driver’s body pose to classify the engaged task [9,10]. The information fusion methods extract both features from the driver’s behaviour (e.g., head or gaze movement) and the vehicle state characteristics to achieve robust task detection [11,12]. Compared with the secondary task detection, the NDA recognition aims to identify the specific activity the driver is engaging in while the vehicle is in the automated driving mode, which could affect the take-over performance. The existing common approaches used in secondary task detection suffer from the limitation of action classification. For instance, during NDA engagement, the driver is free from controlling the vehicle. The maneuver-based methods are therefore not able to capture the driver’s distraction. It is also a challenge to refine activity recognition using a single gesture or gaze modelling method due to the diversity and uncertainty of NDA engagement [13].

Previous studies of drivers’ take-over performance claimed that a sufficient take-over interval for the driver should be between 5 and 8 s [6,14]. The factors that influence the performance include the driver’s state, such as gender, age, and driving experience [15,16], the complexity of the driving scenario [17,18,19], the modality of the take-over request [14,20,21], and the NDAs that the driver engages in [14,22]. In recent years, the effect of NDAs on the take-over transition process has been broadly investigated. Yooh et al. [14] investigated drivers’ take-over performance for three types of NDA, namely phone conversation, smartphone interaction, and video watching tasks, while Zeeb et al. [23] examined the impact of writing an email, reading news, and watching video clips. Results from both studies suggested that NDA engagement can significantly affect take-over quality based on the statistical analysis. One of the limitations of existing studies [24,25] is that NDAs were investigated specifically and independently. When considering a new NDA, such a system needs to conduct the evaluation process again, which limits the extendibility of the driver monitoring or take-over assistance system. There is a lack of systematic methods to group NDAs which could have a similar level of impact on the take-over performance for enhanced scalability. On the other hand, the existing literature concerning the impact of NDAs is normally from the perspective of the driver’s workload [6,26,27]. Situation awareness before take-over is also considered as a crucial factor of safe take-over transition but has not been discussed in association with NDAs [28]. There is a knowledge gap concerning the implication of situation awareness on the take-over process.

It has been stated in the existing literature that the type of NDA that drivers engage in affects their take-over. For instance, compare to auditory related activities, visual related activities lead to a longer reaction time [29]. However, the existing research only focused on a specific visual related task and an auditory related task. There is no further discussion on more different visual related or auditory related activities. Following the survey undertaken by Sivak and Schoettle [30], the identified common NDAs are reading, texting, working, watching movies, and playing games. Since all these common activities are visual related, in this study, we focus on this kind of NDA specifically. The four selected types of NDA include playing games, answering questionnaires, watching videos, and reading news. The device that the driver used to engage all these NDAs is a tablet. Based on the type of interaction between the driver and tablet, the selected NDAs are divided into two groups: active interaction mode and passive interaction mode. Playing games and answering questionnaires are considered as the active interaction mode since the driver and the object respond to each other’s action over time during the engagement. Under the passive interaction mode, e.g., reading news or watching videos, the driver only receives information passively. For simplicity, the active NDAs and passive NDAs will be used to represent the NDAs in the active interaction mode and passive interaction mode, respectively. This study hypothesizes that the workload and demanded attention are different between these two modes, which will lead to different take-over performance.

This paper proposes a two-feed, computer vision-based framework for NDAs/DAs recognition with 3D convolutional neural network (CNN). The driver’s behaviour, including head movement and hand movement, is considered as the input of the CNN framework. Then, the implication of the recognized NDAs in both interaction modes on the take-over performance was investigated. Additionally, based on the captured head movement video, the driver’s road-checking behaviour has been extracted, which is considered a factor that reflects the driver’s situation awareness. If more road-checking behaviour has been performed during the engagement of NDAs, the driver will have more awareness of the surrounding environment. The motivation for performing such behaviour associated with each NDA has been inferred for further understanding the attention demand under different NDAs. The haptic feedback torque was implemented in the steering wheel to support the driver in the control transition. The haptic feedback assistance during the take-over process has been broadly investigated regarding the design of the HMI [31,32,33], especially the steering wheel implemented system [34,35,36]. This study also evaluated its impact and effectiveness in the take-over process.

The paper is organized as below. The NDA detection and recognition system, the experiment design, and the vehicle setting are introduced in Section 2. In Section 3, the performance of the 3D CNN model used in this study is evaluated. Furthermore, the driver’s road-checking behaviour and their take-over performance of each NDA are presented and analysed from both the perspective of the group and individual. The discussion and conclusions are further provided in Section 4.

## 2. Methodology

### 2.1. NDA Detection and Recognition System

The driver’s activities inside the cabin can be divided into 2 groups, which are driving-related activities and non-driving related activities. Gaze estimation is one of the most commonly used methods for the detection of driving-related activities, such as road checking, wing mirror checking, and rear-view mirror checking [37,38,39]. This method focuses on modelling through the driver’s facial features. It can work effectively in detecting the driver’s road-checking behaviour and identifying some activities, e.g., centre console checking and dashboard checking. However, for the application on NDA recognition, such a method can only identify which object (e.g., phone or tablet) that the driver is gazing at but cannot recognise the specific task [40], such as whether the driver is watching videos or playing games with this object, which could lead to different take-over performances [6,23]. Yang et al. [41] proposed a two-stream CNN model for NDA recognition based on the driver’s hand movement. The spatial stream demonstrates high performance on the classification of objects (phone and tablet). The temporal stream uses a stack of optical flow frames which represent the hand movement between 2 RGB frames. Xing et al. [42] further extracted the driver’s body from the RGB frames and used the segmented body frames as the input of the CNN model to recognize the driver’s behaviors. Both methods are based on the 2D CNN model, in which the convolutions only consider the features from the spatial dimension of the frames [43]. The features of hand movement behaviour in a time duration provided in the optical flow stack can only be processed as multiple channels in the spatial dimension. It lacks a direct representation of the motion information in the temporal dimension.

**Figure 1:**
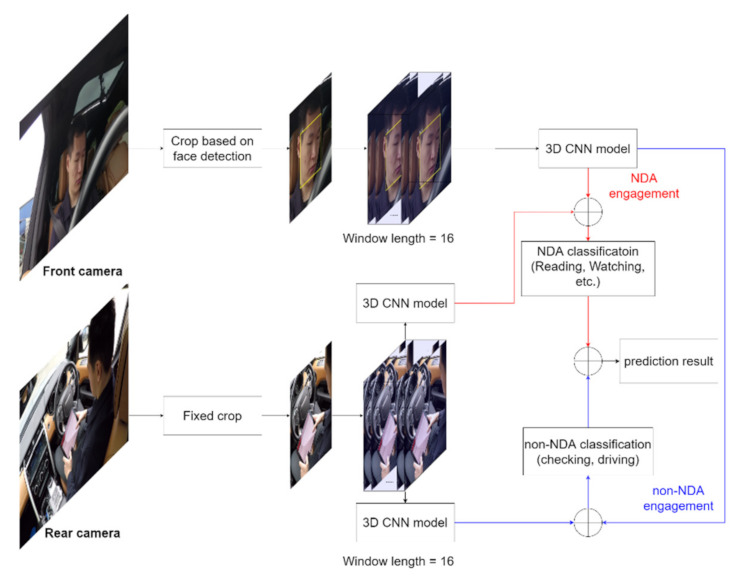
The proposed 2-feed NDA recognition framework.

To address the above problem, this paper introduces a 2-feed 3D CNN-based NDA recognition framework, the flowchart of which is shown in Figure 1. This framework is used to recognize 4 types of NDAs and 2 types of driving activities (DAs). The details of these activities will be introduced in the section below. The front camera captures the driver’s head movement, the image of which is cropped based on the face location automatically. A stack of head movement frames within a certain time window, denoted as d, are inputted to a 3D CNN model to detect whether the driver is performing NDAs or DAs. The rear camera focuses on the driver hand movement. The frame is fixedly cropped since the hand movement is limited in the vehicle cabin. A stack of hand movement frames within the same time window is imported into 2 separate models to further identify the specific NDA or DA. The final prediction result is obtained from the concatenated results of the NDA and DA classification.

**Figure 2:**
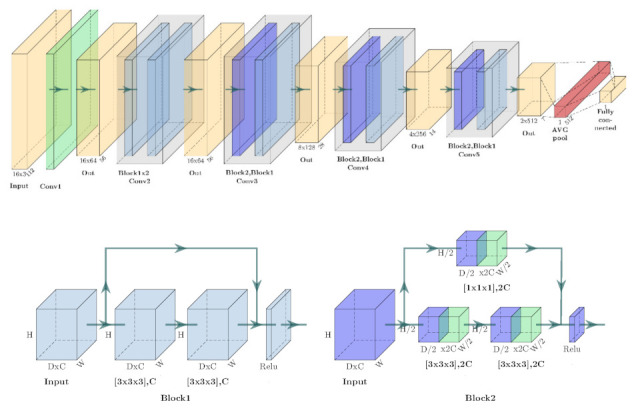
The proposed 3D CNN model. There are two types of residual block in this network, which are detailed in the bottom graph and indicated as different colours.

The architecture of the network is based on 3D ResNet-18, whose capability in terms of video recognition has been proven in [44,45]. The structure is illustrated in Figure 2. The size of the input frame stack and the feature map is notated as c×d×h×w, where c is the number of the channels, d is the depth of the input (the number of frames in the time window for this case), h is the height of the frame, and w is the width of the frame. The convolutional kernel size is denoted as $d_{k}\times k\times k$, where $d_{k}$ is the depth of the kernel and k is the spatial size of the kernel. The size of the cropped video clips for both feeds is 400×400 pixels, which is resized to 120×120 pixels and then randomly cropped to 112×112 pixels. A total of 16 frames in a clip are used as an input of the network for training, which can be denoted as 3×16×112×112. A total of 5 groups of the convolutional layer are used in the network. The size of the convolutional kernel and the extracted feature map from each layer are presented in Figure 2. There are 2 types of residual blocks used in the last 4 convolution layer groups, which are shown at the bottom of Figure 2. The shortcut structure of each block can be expressed as:

(1) $$x_{l+1}=\;F\left(x_{l},w_{l}\right)+x_{l}$$

where $x_{l}$, $w_{l}$ are the input and weights of the convolutional layer l respectively. $F\left(x_{l},w_{l}\right)$ represents the function where the residual mapping is learned. Batch normalisation (BN) is employed in each convolution layer.

For the training process, Kaiming initialization [46] is applied for weight initialization. The initial learning rate is set as 0.001, which is dynamically reduced when the validation loss stops improving. The loss of a prediction output x in this network can be described as:

(2) $$Loss\left(x,label\right)=w_{c}\left[label\right]\left(-x\left[label\right]+\log\left(\underset{j}{\sum }e^{\left(x\left[j\right]\right)}\right)\right)$$

where $w_{c}$ is the weight distribution of the classes in the dataset to improve the data imbalance in a mini-batch; label is the true class of the instance; j is the index of all classes. The losses are averaged for each mini-batch.

The prediction probability of NDA detection based on the driver’s head movement is denoted as $P_{d}$, which has only two states: DA engagement and NDA engagement, denoted as $c_{d}$ and $c_{N}$, respectively. The prediction probability for these two classes is therefore presented as $P_{d}\left[c_{d}\right]$ and $P_{d}\left[c_{N}\right]$. Two different 3D CNN models have been trained for DA and NDA classification based on hand movement. The prediction probability for these 2 models are denoted as $P_{dc}$ and $P_{Nc}$.

The final prediction scores for all NDA and DA classes, denoted by S, can be expressed as:

(3) $$S=S_{d}\cup S_{N}$$

where $S_{d}$ and $S_{N}$ are the final scores of the DA classification and NDA classification, respectively. The score of a single DA can be expressed as:

(4) $$S_{d}\left(i_{d}\right)=P_{dc}\left(i_{d}\right)P_{d}\left[c_{d}\right]$$

where $i_{d}$ is the index of the DAs.

The score of a single NDA can be expressed as:

(5) $$S_{N}\left(i_{N}\right)=P_{Nc}\left(i_{N}\right)P_{d}\left[c_{N}\right]$$

where $i_{N}$ is the index of the NDAs.

### 2.2. Experiment Design

**Figure 3:**
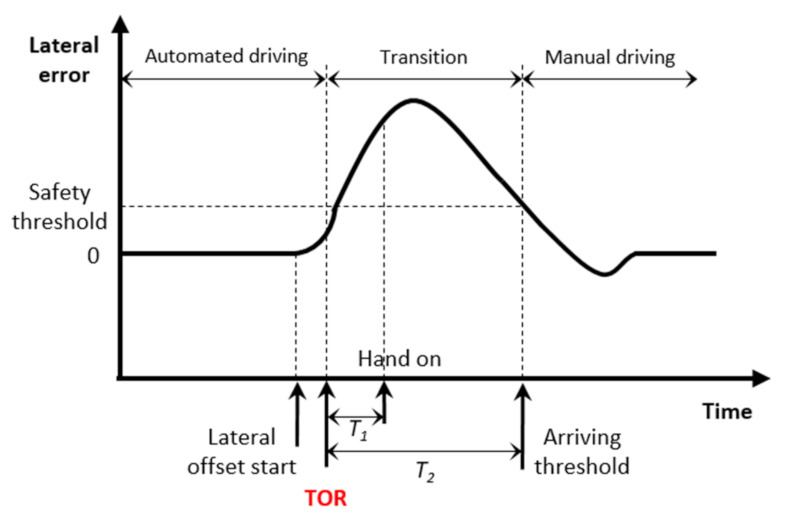
Concept of the take-over process.

Take-over process: Figure 3 plots the design of the take-over process in a trial. During a trial, the vehicle was driving automatically initially while the participant was required to engage in a certain type of NDA or check the road. Then, the take-over process started after a lateral offset was implemented to the vehicle. The lateral error is defined as the distance between the vehicle position and the closest point on the path. After a lateral offset is implemented, the vehicle is in an improper position on the road. Then, an acoustic signal was given to the participant as a take-over request (TOR), which requests he/her to take control of the vehicle and bring it back to the right position. In Figure 3, $T_{1}$ indicates the time needed for the driver to put her/his hand on the steering wheel. To achieve a safe and smooth take-over transition, a haptic torque was implemented to help the driver guide the vehicle to the reference route. The haptic torque was engaged as soon as the driver applies the torque to the wheel and gradually fades away. After the lateral error achieves the maximum value, the vehicle returns to the reference route. A threshold of the safety distance is defined, which indicates that the control transition is finished, and the driver could achieve safe manual driving afterwards. In this study, the threshold was set as 0.7 m, which is the maximum lateral error to keep the vehicle inside the lane. In Figure 3, $T_{2}$ refers to the time needed from TOR to the time when the vehicle arrives at the threshold, which is considered as a criterion to evaluate the take-over performance in this study.

**Figure 4:**
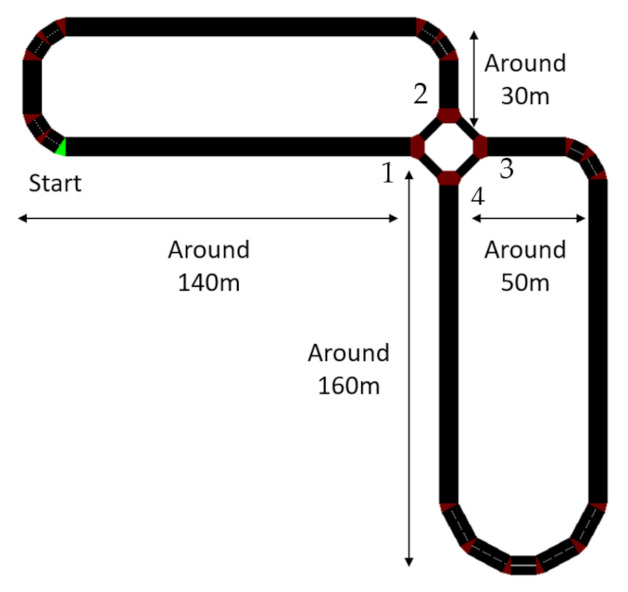
Sketch map of the track.

Track and take-over scenarios: The testing track is a two-lane road with a mini roundabout, as shown in Figure 4. The start point is highlighted with green colour. In the odd loop, the vehicle enters from 1 into the roundabout and leaves from 3. Then, it enters from 4 and leaves from 2. In the even loop, the vehicle enters from 1 and leaves the roundabout from 4. Then, it enters the roundabout from 3 and leaves from 2. The TOR signal was issued at specific points on the track to avoid the area around the roundabout for safety concerns. The lateral offset was set as 1.5 m with a small variation in the real trials. The maximum speed of the vehicle was set as 30 mph. The interval between TORs was randomly selected from the range of 5–9 min.

NDAs and DAs: In this study, 4 types of NDA and 2 types of DAs were investigated. A tablet was used for the engagement of NDAs, which include reading news, watching videos, playing games, and answering questionnaires. For reading news, the participants were required to read some articles from BBC News. For watching videos, the participants were asked to watch some videos from YouTube. For playing games, the participants were required to play Temple Run. For the NDA of answering questionnaires, the participants were required to complete a questionnaire, which comprised some objective and subjective questions about this experiment. The DAs considered in this study are road checking and driving. In the experiment, each participant completed 7 trials, including 4 trials for 4 types of NDA respectively and 1 trial of watching the road with no NDA engagement. For the remaining 2 trials, 2 activities were randomly selected from the 5 activities mentioned above. The order of activities for each participant was randomized.

Participants: 14 participants (12 male and 2 female; aged 24–30) participated in this experiment. A valid UK driving license was required. None of them had driving experience with high-level automated driving vehicles.

**Figure 5:**
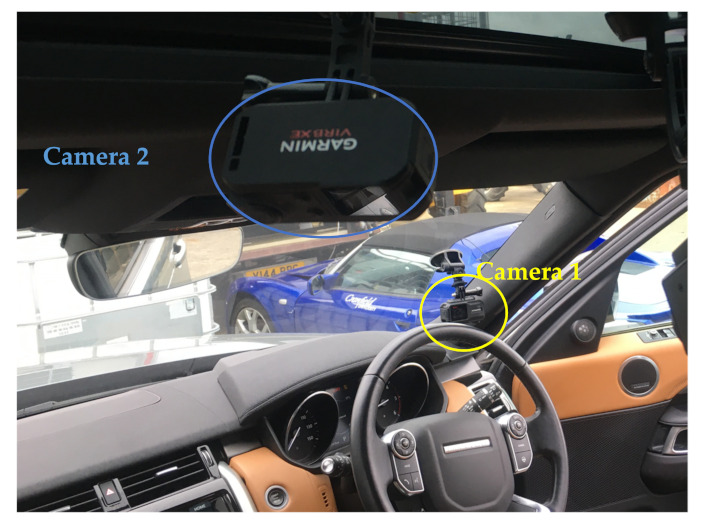
A illustration of the two cameras inside the vehicle.

Video acquisition and dataset pre-processing: 2 Garmin Virb Action Cameras were used to capture the driver’s behaviour inside the vehicle cabin in this experiment. Both cameras had a spatial resolution set at 1920×1440 pixels and a temporal resolution set at 30 frames per second (fps). As shown in Figure 5, Camera 1 extracts the driver’s head movement and facial information and detects if the driver is checking the road or engaging with NDAs. Camera 2 captures the driver’s hand movement when interacting with the tablet or the primary steering wheel, which was mounted on the vehicle’s roof between two front seats.

In the dataset for the NDA recognition framework, a single instance, denoted by I, contains a pair of synchronised frame stacks ($I_{f}$, $I_{r}$) from Camera 1 and Camera 2, respectively. The recorded video from each camera was split into several clips. There are 48 frames in each clip, where 16 adjacent frames were randomly picked and used as an input instance $I_{f}$ or $I_{r}$. A total of 3624 instances for 6 classes were extracted from the videos of the 14 participants. Specifically, for the 2 DAs, the driving instances were extracted from the videos of the take-over process at the end of an NDA trial. The road-checking instances were extracted from the videos of the watching road trial and the driver’s road checking behaviour in an NDAs trial. For every participant, there are around 40 instances for each NDA class and each DA class. The data of 10 participants were randomly selected and used for training, the data of 2 participants were used for validation, and the remaining 2 participants were used for testing. In summary, a total of 2598, 467, and 559 instances were used for training, validation, and testing, respectively.

### 2.3. Vehicle Setting

**Figure 6:**
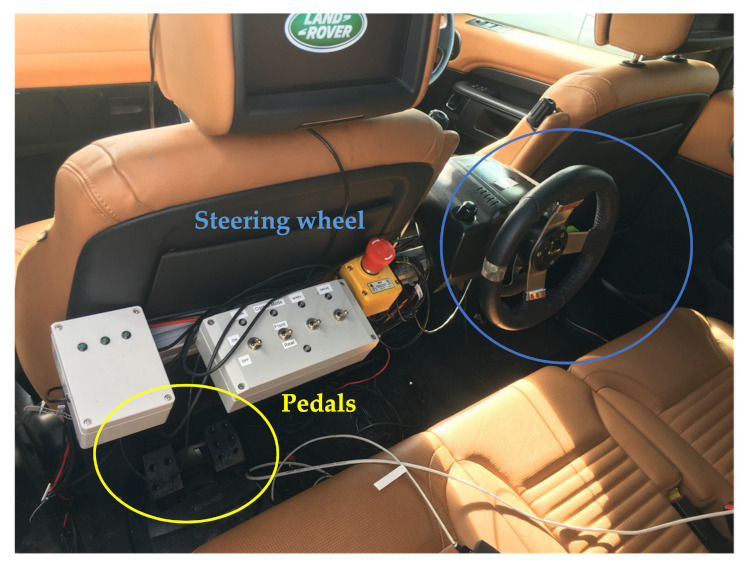
A view of the modified vehicle, where the rear steering wheel and pedal have been highlighted.

Vehicle Modification: An instrumented Land Rover Discovery 5 was employed as the testing bed, which was modified to accommodate both autonomous and human driving. An electric motor, operating on the steering column, was used for steering and another electric motor was used to control the throttle pedal position. Braking was modified using a pneumatic actuator on the brake pedal. To ensure safety, a second steering wheel and a set of pedals were added in the back seat, shown in Figure 6, which allows a safety driver to intervene and override the autonomous system. For the path following, the pure pursuit algorithm was used to generate the reference steering angle. The rear steering wheel was controlled using the reference steering angles and the front wheel follows the rear wheel.

Vehicle control and data acquisition: The OXTS RT1003 (Oxford Technical Solutions Limited, Oxfordshire, United Kingdom) with RTK GPS system provides the global vehicle position with an accuracy of 2 cm and the heading angle with an accuracy of less than 1 degree. The data of vehicle status were recorded in the Microautobox I (dSPACE, Paderborn, Germany) at a sampling rate of 1 k Hz. The data include driver steering torque, autonomous steering torque, vehicle position and heading, vehicle velocity, steering angle, and take-over signal. The path was recorded beforehand at a sampling rate of 1 k Hz and then resampled by the linear interpolation to a spatial accuracy of 0.2 m.

**Figure 7:**
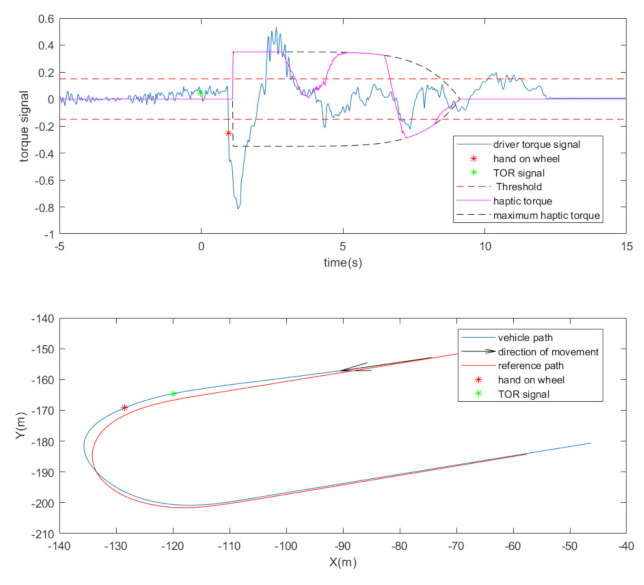
Top plot presents the driver’s torque and the haptic torque for 1 instance. Bottom plot presents the corresponding vehicle movement in the track.

A certain torque threshold was applied for hand-on-wheel time ($T_{1}$) detection. It was experimentally determined to avoid false take-over detection due to sensor noise. An instance of the driver’s torque during a take-over process is shown in the top plot of Figure 7. The corresponding vehicle route is presented in the bottom plot of Figure 7.

After the driver takes control of the steering wheel, the vehicle provides haptic cues to the driver, in the form of torque on the steering wheel, to increase the driver’s awareness of the environment. The haptic decays over a certain amount of time and eventually reaches 0 to give the driver full control. The value of the torque is calculated using

(6) $$\tau_{haptic}\left(t\right)=K_{t}\left(t\right)K_{p}\left(\delta -\delta_{ref}\right)\;$$

where δ is the vehicle steering angle; $\delta_{ref}$ is the reference steering angle calculated by the path following algorithm; $K_{p}$ is a constant gain, and $K_{t}\left(t\right)$ is a decaying gain which is a function of time starting from 1 and reaching 0 at the end of the take-over period. The decaying profile is represented as the black dot line in the top plot of Figure 7. The decaying duration chosen for this experiment was 8 s. The torque value is normalized between −1 and 1, where 1 indicates the maximum torque of the electric motor in one direction and −1 indicates the maximum torque in another direction. The maximum amplitude of the torque was a tuning parameter. Each participant tried two of three pre-set values randomly in the trials: 0.35, 0.45, 0.55.

## 3. Results

### 3.1. Activity Classification

**Figure 8:**
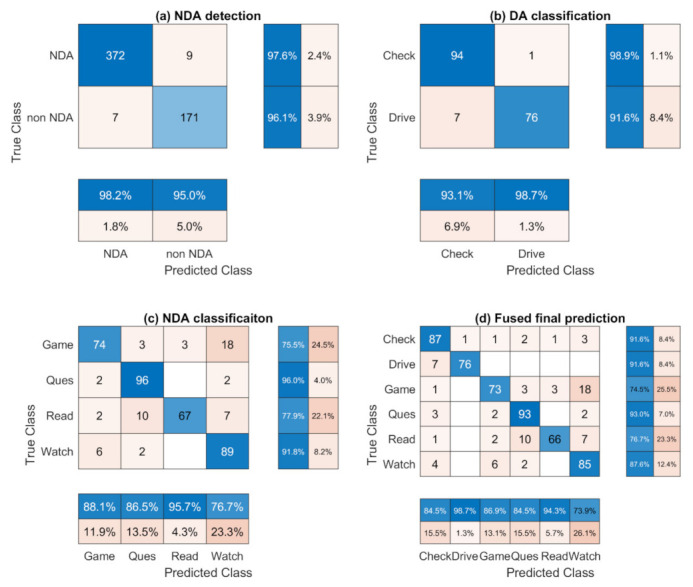
Confusion matrices of the 3 developed 3D CNN models and the fused results. The precision and recall for each class are presented in the bottom and right of the figure, respectively.

The confusion matrices of the proposed two-feeds 3D CNN models are presented in Figure 8. All six activities are abridged as a term for the convenience of result presentation. Check and Drive refer to two DAs, which are road checking and driving. Game, Ques, Read, and Watch refer to the four kinds of NDA, which are playing games, answering questionnaires, reading news, and watching videos, respectively. For the NDA detection based on the driver’s head movement (Figure 8a), the precision and recall of both classes are over 95%. The accuracy of the NDA detection is 97.14%, as shown in Table 1. The DA classification (Figure 8b) also shows a high precision and recall (>90%) and the accuracy is 95.51% (Table 1). A total of 8.4% driving instances were misclassified as road checking, which is due to the hand gesture where sometimes the driver holds the tablet in one hand and controls the steering wheel with another hand during the take-over process. As shown in the confusion matrix for NDA classification (Figure 8c), among all these NDAs, answering questionnaires achieves the best performance (86.5% for precision and 96.0% for recall). The lowest recall value is from playing games, which is 75.5%. The main contribution of the false negative is from watching videos, which is due to the limited hand movement in some game engagement instances, and comparing with the other two NDAs, watching videos has similar spatial information with playing games. The highest value of precision is from reading news (95.7%), while the recall is only 77.9%. Answering questionnaires and watching videos are the main contributions of the false negative. The prediction of reading news could be tricky, because sometimes the limited hand gesture change could lead to confusion with watching videos. Sometimes, the frequent movement required for turning pages could be misclassified as answering questionnaires since the spatial background could be similar between these two classes. From the final fusion matrix, shown in Figure 8d, it can be observed that the driving class achieves high values in both precision and recall. Since the results are obtained by combining the 3 models, the value of each class shows a similar trend with the value in the separate model mentioned above. The total accuracy of the final prediction is 85.87%.

### 3.2. Road-Checking Behaviour Analysis

The road-checking behaviour performed during the NDA engagement has been extracted by the proposed NDA recognition framework and shown in Table 2. The checking period is determined as the division of the total time duration of the NDA engagement trials by the total number of instances of road-checking behaviour in these trials. The motivation of the road-checking behaviour is manually inferred from the recorded videos, which has been divided into four classes, which are bumping, approaching junctions, breakpoint, and other. Bumping refers to the checking behaviour caused by the vehicle vibration that happened on the uneven road surface. Approaching junctions refers to the checking behaviour whereby the driver glanced as the vehicle approached the roundabout and turning. For breakpoint, it indicates the checking behaviour, which is performed in a short break during the NDA engagement. For example, the driver could check the surrounding environment after she/he finished watching a short clip or a round of the game. Other includes the motivations which are different to the above-mentioned class, such as illumination changing or regular checking, etc.

From Table 2, it can be seen that the checking period of watching videos is the shortest (37.10 s). The period of reading news is slightly higher at 51.64 s. As the passive NDAs, both NDAs show a similar proportion of motivation. Approaching junction is the main motivation, which is above 50%. Playing games shows a relatively longer checking period (79.13 s) than the passive NDAs, where breakpoint (59.04%) is the dominant motivation. Answering questionnaires has the least road-checking behaviour, mostly once or twice during a single trial. The motivation shows a similar trend with the passive NDAs, but with a higher proportion of the breakpoint (13.64%). For the passive NDAs, the driver performs more frequent road-checking behaviour compared to the active NDAs. This suggests that the driver pays less attention to the engagement under this type of NDAs. The main motivations are approaching junctions and bumping. This suggests that the driver is more sensitive to the change of the vehicle state, such as velocity change, vibration, or turning. Such checking behaviour is important for safety control transition if the TOR is given when the vehicle state is changing. The results of the active NDAs engagement show that the driver performs much less road-checking behaviour, particularly for the engagement of answering questionnaires. For playing games, the checking behaviour mainly happens in breakpoint, which suggests the driver is at a high attention level and not sensitive to the environment change during the engagement. Therefore, for the passive NDA, achieving a high-quality control transition could be more challenging for the driver due to the limited situational awareness.

### 3.3. Take-Over Performance

**Figure 9:**
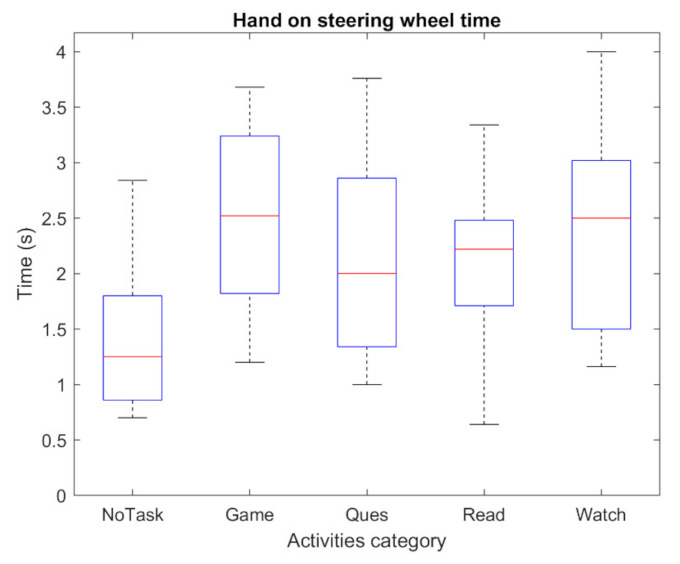
The hand-on-wheel time performance. NoTask refers to the performance in watching road trial.

**Figure 10:**
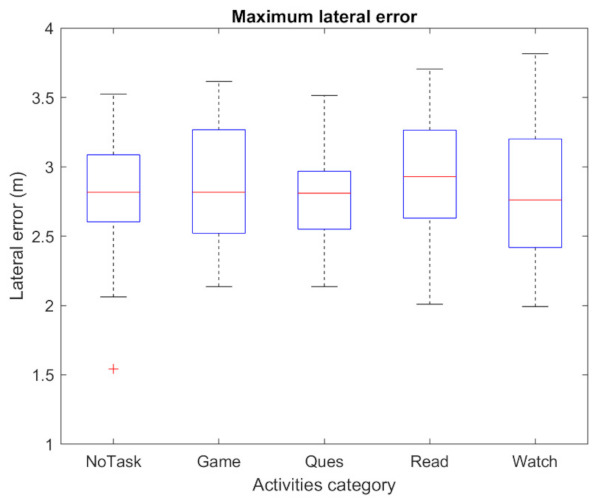
Maximum lateral error achieving. An outlier is presented with the plus sign which represents a high concentration level of the driver during the road checking task.

**Figure 11:**
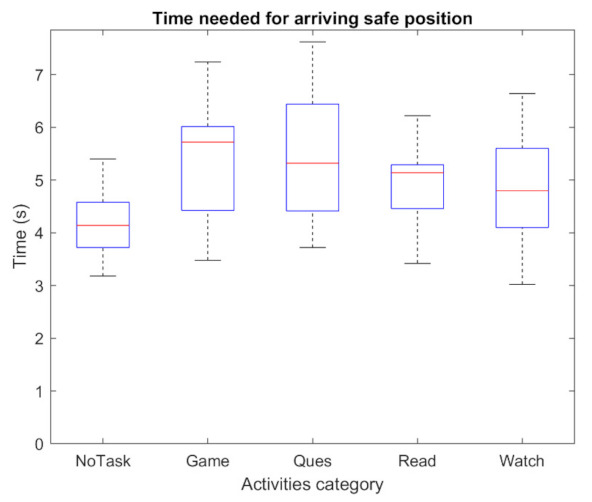
Time cost for the vehicle back to the safe position.

The take-over performance is presented and evaluated in this section. Figure 9 shows the driver’s hand-on-wheel time ($T_{1}$) against five activities. The shortest mean value of $T_{1}$ is around 1.3 s, which is NoTask. For the remaining four NDAs, the average value is in the range of 1.9–2.6 s. Answering questionnaires shows a relatively shorter $T_{1}$ than other NDAs. The maximum lateral error is presented in Figure 10. It can be observed that the value for each NDA and NoTask are similar, the average value is around 2.8 m. In the experiment, the TOR signal was given after the lateral error achieved 1.5 m, while most of the drivers could control the vehicle within a maximum 3.5 m lateral error. However, the impact of the NDAs engagement on the take-over performance mainly presented after the maximum lateral error was achieved. From Figure 11, the baseline of time needed to achieve the safe position ($T_{2}$) is around 4.16 s for NoTask. The $T_{2}$ for NDAs engagement is at least 0.5 s more, which suggests the engagement of NDAs could increase the time that the vehicle stays in the dangerous position on the road. The mean and standard deviation of $T_{2}$ for each activity are presented in Table 3. For the passive NDAs, the mean values of watching videos and reading news, as the passive NDAs, are 4.74 s and 4.96 s respectively, which are higher than the active NDAs, answering questionnaires and playing games (5.45 s and 5.43 s respectively). The standard deviation of the NDAs is higher than the NoTask, which suggests higher individual differences in terms of take-over performance in NDAs engagement. From Figure 9 and Figure 11, it can be observed that the driver under active NDAs engagement requests more time to drive the vehicle back to the safe position, which suggests a higher mental demand or workload during such NDAs engagement. After receiving the TOR signal, the driver needs more time to build awareness of the surrounding environment and it is more challenging for them to switch to the take-over process from the NDA engagement.

Table 4 presents the $T_{2}$ under different levels of haptic feedback. For a low level of haptic torque, the mean value of $T_{2}$ is 5.32 s, which is the lowest among all the evaluated levels. The standard deviation is 1.12 s, which suggests that all the participants have higher tolerance at this level of haptic torque assistance. It can be seen that the increase of the torque level could result in the decrease of the mean value of $T_{2}$, which means a higher level of haptic torque could support the driver to reduce $T_{2}$ and improve their take-over performance. However, the standard deviation increases (1.55 s for medium level and 1.32 s for high level). This suggests that some of the participants could distrust and resist the higher level of haptic torque and take a longer $T_{2}$.

## 4. Conclusions

Achieving a safe control transition is one of the most important challenges in level 3 automation systems and is influenced by many factors, where the driver’s mental state and driving-environment awareness before take-over play an important role. We proposed a two-feed 3D CNN-based NDA recognition system which can automatically detect and classify the driver’s NDAs engagement and DA activities with high accuracy. It has been demonstrated that both head and hand movement are crucial for achieving this target. This study further investigated the implication of the NDAs engagement on both perspectives of roading checking behaviour and take-over performance. Based on the investigation, a category method has been proposed to group the NDAs, which aims to extend the application of this study on a wide range of NDAs. Moreover, the effectiveness of the steering wheel haptic assistance system for the take-over process has been evaluated.

For the investigation of road checking behaviour, the driver always performs such behaviour during the engagement of NDAs to ensure driving safety. There is less road checking behaviour under the active NDAs engagement. The motivation study shows the driver mainly checks the road in the breakpoint and is less sensitive to the change of the vehicle state, which suggests that the driver paid more attention to the activity and has less awareness of the driving environment. Since the lack of observation could be dangerous, the driver should be reminded to monitor their surroundings to improve situation awareness when they engage in this kind of NDA for a long period without road-checking behaviour. From the take-over performance point of view, the engagement of NDAs leads to a negative effect (longer $T_{2}$). The engagement of active NDAs could demand even more time. Furthermore, haptic torque assistance could improve the take-over performance, as evidenced by decreasing $T_{2}$. However, a higher level of haptic torque could result in the driver’s resistance.

In summary, this investigation helps us develop a deeper understanding of the implication of the driver’s behaviour on the control transition in conditional automation, which further helps the design of HMI and take-over strategies to accomplish a safe take-over. The type of NDA determines the level of the driver’s mental demand, which further affects their situation awareness and take-over performance. The observed results also suggest that the take-over process could benefit from highly frequent road-checking and haptic feedback assistance. The existing HMI design only considers the type of NDAs in the take-over process from the perspective of the take-over request modality. From this study, the road-checking behaviour of the driver during NDAs engagement also matters for reducing the take-over time. An alert system for checking the surrounding environment should be considered for further HMI design.

## Figures and Tables

**Table 1:** Performance of 3D CNN model.

Term	NDA Detection	DA Classification	NDA Classification	Final Prediction
Accuracy	97.14%	95.51%	85.56%	85.87%
Weighted F1 score	97.14%	95.49%	85.46%	85.88%

**Table 2:** Road-checking behaviour evaluation.

NDAs	Checking Period (s)	Percentage of Checking for Corresponding Motivation			
		Bumping	Approaching Junctions	Breakpoint	Others
Watching videos	37.10	19.88%	52.05%	5.85%	22.22%
Reading news	51.64	16.78%	51.75%	7.69%	23.78%
Playing games	79.13	3.61%	26.50%	59.04%	10.84%
Answering questionnaires	123.00	18.18%	50.00%	13.64%	18.18%

**Table 3:** Time to threshold for all activities.

Time to Threshold	Activities				
	No Task	Watch	Read	Ques	Game
Mean (s)	4.16	4.74	4.96	5.45	5.43
Standard deviation (s)	0.67	1.12	0.87	1.23	1.14

**Table 4:** Time to threshold for different haptic torque levels.

Time to Threshold	Haptic Torque Level		
	Low	Medium	High
Mean (s)	5.32	4.97	4.83
Standard deviation (s)	1.12	1.55	1.32
